# A Comparative Study of Health Disparities in Cervical Cancer Mortality Rates Through Time Between Black and Caucasian Women in Alabama and the US

**DOI:** 10.20849/ijsn.v6i1.864

**Published:** 2021-03-04

**Authors:** Ehsan Abdalla, Tsegaye Habtemariam, Souleymane Fall, Roberta Troy, Berhanu Tameru, David Nganwa

**Affiliations:** 1 Department of Graduate Public Health, College of Veterinary Medicine, Tuskegee University, USA; 2 Department of Pathobiology, College of Veterinary Medicine, Tuskegee University, USA; 3 College of Agriculture, Environment and Nutrition Sciences, Tuskegee University, USA; 4 Interim Provost & Vice President for Academic Affairs, Office of the Provost, Tuskegee University, USA

**Keywords:** cervical cancer, mortality rate, pap smear, healthy people 2020

## Abstract

**Background:**

The main purpose of this study was to assess changes in cervical cancer mortality rates through time between Black and Caucasian women residing in Alabama and the US.

**Methods:**

Alabama cervical cancer mortality rates (MR), percentage differences, percentage changes and annual percentage changes for trends were compared with the US baseline and target rates. The US Baseline data and target objectives of utilization of cervical cancer screening and MR were obtained from Healthy People 2020. The cervical cancer behavioral risk factors and utilization of screening tests data were obtained from CDC’s Behavioral Risk Factor Surveillance System (BRFSS). The cervical cancer MR data were obtained from the Surveillance, Epidemiology, and End Results (SEER). The analysis was done using SEER*Stat and Linear Trendlines analysis.

**Results:**

Although Blacks in Alabama had higher cervical cancer MR through times, a decreasing trend was noted for both races. However, in Alabama, there is no significant change in Blacks aged 65 years and older in cervical cancer MR, despite a high screening rate compared to Whites. In contrast, between 2002 and 2012, Whites in Alabama and the US made a significant progress toward the Healthy People 2020 goal.

**Conclusions:**

In Alabama, there exists cervical cancer MR disparity in Blacks despite the higher rates of screening for cervical cancer as would otherwise be expected. The state has not yet achieved the Healthy People 2020 goal. Public health officials should monitor progress toward reduction and/or elimination of these disparities by focusing in a follow up of screening.

## 1. Introduction

Worldwide, cervical cancer (CerCancer) is the third most common cancer among women and the second most frequent cause of cancer-related deaths, accounting for nearly 300,000 deaths annually. CerCancer is the one of the most common cancers affecting the US women and ranks 14th in frequency (National Cancer Institute (NCI), 2010). CerCancer, caused by particular types of human papillomavirus (HPV) is a preventable cancer because of its long evolving period where it can be detected early by screening process (Tabatabai MA, Kengwoung-Keumo J-J, Eby WM, Bae S, & Guemmegne JT, 2014). It is also a highly treatable disease because if detected early by the Papanicolaou test (Pap test), a simple procedure in which a small sample of cells is collected from the cervix and examined and, if it is at a localized stage, the 5-year survival rate for invasive cervical cancer is 91% (Center for Disease Control and Prevention (CDC), 2015). This means that if all women complied with screening, treatment and follow-up recommendations, almost all cervical cancer deaths could be avoided (American Cancer Society, 2015b). In 2013, the National Breast and Cervical cancer Early Detection Program (NBCCEDP) screened 208,682 women for cervical cancer with the Pap test and diagnosed 252 cervical cancer and 9,505 premalignant cervical lesions, of which 36% were high-grade (Center for Disease Control and Prevention (CDC) & National Association of Chronic Disease Directors, 2016).

The US Preventive Services Task Force (USPSTF) recommends screening for cervical cancer in women aged 21–65 years with cytology (Pap smear) every 3 years. For women aged 30 – 65 years who want to lengthen the screening interval, screening with a combination of cytology and HPV testing every 5 years is recommended (U.S. Preventive Services Task Force, 2008). Since screening programs using the Pap test were implemented widely more than 50 years ago, cervical cancer deaths have declined by 75% nationwide. Yet cervical cancer still takes the lives of approximately 4,000 women in the US each year (Freeman HP & Wingrove BK, 2005). Furthermore, racial disparities exist in both incidence and mortality rates although there is widespread use of screening (Pap smears and HPV DNA testing) cervical dysplasia (precancerous changes). Accordingly, cervical cancer deaths are higher among Hispanic and Blacks relative to Whites. As a result even though Blacks have experienced the largest decreases in mortality rates since 1992 they still have rates more than double that of Whites (American Cancer Society, 2015a).

Disparities may be influenced by the geographic distribution of minority groups in the US, depicting an urban-rural gradient (i.e., mortality lower in urban areas, higher in rural areas) reflecting less access to care and poorer outcomes, though this has not been demonstrated clearly (H. P. a. W. Freeman, B.K., 2005). Women who live in largely rural and suburban counties in the states including the Deep South (Alabama and Mississippi), have consistently higher rates of cervical cancer mortality than do women in other parts of the country. Data on Blacks in the Deep South illustrate this situation clearly. Cervical cancer mortality in Alabama was estimated at 3.0 per 100,000 population in 2012. This rate was slightly higher than the US rate of 2.4 per 100,000 population (Alabama Statewide Cancer Registry (ASCR) & Alabama Department of Public Health, 2012). On the other hand Alabama Statewide Cancer Registry data indicates that on average the cervical cancer mortality rates during 2001 – 2010 for Black women was 5.3 per 100,000 population compared to 2.4 per 100,000 population in Whites (Alabama Statewide Cancer Registry (ASCR) & Alabama Department of Public Health, 2012). Nevertheless, mortality rates have been steadily decreasing with the advent of improved cervical cancer screening/treatment modalities. A primary focus in modeling cervical cancer mortality rates is therefore the disparity of this screening/treatment utilization among different races (Tabatabai MA et al., 2014).

One of the overall Healthy People 2010 initiative objectives for the cervical cancer screening was to increase the proportion of women who receive a Pap test a) by increasing the screening rate to 97% by 2010, for women aged 18 years and older who have previously received a Pap test and b) by increasing the screening rate to 90% by 2010, for women aged 18 years and older who received a Pap test within the preceding 3 years (Table 2). The overall objective for the cervical cancer mortality was to reduce the death rate to 2.0 deaths per 100,000 populations (Table 1) (C. f. D. C. a. P. (CDC), 2010b). With regard to treatment, compared to Whites and Blacks were more likely to have received or planned to receive chemotherapy (15.9%; vs 26.3%); were more likely to undergo surgery only (40.1% vs 27.6%); and were more likely to receive radiation only (30.8% vs 39.4%). Furthermore, slightly more Blacks had neither surgery nor radiation (8.5% vs 10.9%) (Movva S et al., 2008 ). The initially established goals to reduce the number of new cases, including illness, disability, and death caused by cancer, through an increased use of preventive measures, had not been met by the year 2010 (C. f. D. C. a. P. (CDC), 2010b). Therefore, in Healthy People 2020 the cervical cancer objectives have been expanded to include a broader range of measures than those presented in Healthy People 2010, reflecting the latest trends in cancer prevention and diagnosis (Table 1). In addition to objectives on mortality, screening, counseling, survival, and cancer registries, the Healthy People 2020 Cancer Topic Area includes new objectives on cancer incidence, quality of life for cancer survivors, among others (C. f. D. C. a. P. (CDC), 2010a). The available data indicate that the cervical cancer mortality among Black Alabamians is consistently high, despite higher screening rate compared to their White counterparts. Therefore, our objective was to assess changes in cervical cancer mortality rates through time between Black and Caucasian women residing in Alabama and the US.

## 2. Materials and Methods

### 2.1 Data Source

Cervical cancer mortality data were collected from the Surveillance, Epidemiology, and End Results (SEER) database for the years 1990 to 2012. Data on behavioral risk factors and use of screening tests were obtained from the National Health Interview Survey (NHIS) conducted by the National Center for Health Statistics (NCHS), CDC’s Behavioral Risk Factor Surveillance System (BRFSS). Data of US baseline and target for measurable objectives C-4 and C-15 (Tables 1 and 2) were obtained from Healthy People 2020. In most cases, the US baseline data provide the point from which Healthy People 2010 and Healthy People 2020 target rates were set (Tables 1 and 2). The 3–11a and 3–11b Healthy People 2010 and C-4 and C-15 Healthy People 2020 objectives regarding Cervical cancer death and screening, in DATA2010 and DATA2020 are described in Tables 1 and 2 (C. f. D. C. a. P. (CDC), 2014).

### 2.2 Analysis

The Percentage differences *(PD)* of women adherence to cervical cancer screening within every three years, in Alabama and the US *(i)* stratified by race *(r)* and age groups *(a,)* were calculated using the mathematical formula:

(1) $$PD_{ira}=\frac{\left(SPB_{ira}-SPf_{ira}\right)}{SPB_{ira}}$$

Where *i = 1,2* is Alabama and the US, *r = 1,2* is Blacks, Whites, *a* is age group (18 & older), *SPb*_*ira*_ = *SPb (L*_*i*_ *E*_*r*_ *A*_*a*_*)* and *SPf*_*ira*_ = *SPf (L*_*i*_ *E*_*r*_ *A*_*a*_*)* are the percentages of women adherence to cervical cancer screening (f = b + 3 years) for geographic area *i,* race *r* and age group *a* respectively.

The Percentage differences *(PD)* of women aged 18 years and older, adherence to cervical cancer screening within every three years, from the Healthy People 2020 US baseline and target rate in Alabama and US *(i)* stratified by race *(r)* were calculated using the mathematical formula:

(2) $$PD_{ir}=\frac{\left(SP_{ir}-B_{ir}\right)}{SP_{ir}}$$

Where *PD*_*ira*_ is percentage difference of women adherence to cervical cancer screening within every three years, *i = 1, 2* is Alabama and the US, *r = 1, 2* is Black, White women respectively, *B*_*ir*_ = *B (L*_*i*_ *E*_*r*_*); 0, 1* is the Healthy People 2020 US baseline and target mortality rate respectively, *SP*_*ir*_ = *SP (L*_*i*_ *E*_*r*_*)* is the percentage of women adherence to cervical cancer screening for geographic area *i,* and race *r* respectively.

SEER*Stat version 8.4.1 (National Cancer Institute (NIH), 2015) in conjunction with Linear Trendlines Analysis to model the racial changes of cervical cancer mortality rates through time in Alabama and the US. Age-adjusted death rates, expressed per 100,000 population, were computed by using the 2000 US standard population in the trend calculations.

The cervical cancer mortality rates *(MR)* in Alabama and US (*i*) stratified by race *(r)* and age group (*a*) was calculated using the mathematical formula *(NCI) (Tabatabai MA et al., 2014)*:

(3) $$MR_{ira}=\frac{\left(NCD_{ira}\right)}{NFP_{ira}}x100,000$$

Where *NCD*_*ira*_ = *NCD (L*_*i*_ *E*_*r*_ *A*_*a*_*)* and *NFP*_*ira*_ = *NFP (L*_*i*_ *E*_*r*_ *A*_*a*_*)* are number of cervical cancer deaths of those who lived in geographic area *L*_*i*_, with race *E*_*r*_ and age group *A*_*a*_ respectively. The indices *i = 1, 2* is Alabama and the US, *r = 1, 2* is Blacks and Whites, *a = 1, 2* is age group 20 – 64 years and 65 years & older respectively.

The percentage differences *(PD)* in cervical cancer mortality rates of Blacks and Whites in Alabama and US *(i)* stratified by age group *(a)* was calculated using the mathematical formula:

(4) $$PD_{ia}=\frac{\left(MRb_{ia}-MRw_{ia}\right)}{MRb_{ia}}$$

Where *MRb*_*ia*_ = *MRb (L*_*i*_ *A*_*a*_*) and MRw*_*ia*_ *= MRw (L*_*i*_ *A*_*a*_*)* are the rates of cervical cancer death for Blacks and Whites and for geographic area *i* and age group *a* respectively, the indices *i = 1, 2* is Alabama and the US, *a = 1, 2* is age group 20 – 64 years and 65 years & older respectively.

The Percentage differences *(PD)* in observed cervical cancer mortality from the Healthy People 2020 US baseline rate in Alabama and US *(i)* stratified by race *(r)* and age a group *(a)* was calculated using the mathematical formula:

(5) $$PD_{ira}=\frac{\left(MRb_{ira}-P20_{ira}^{l}\right)}{MRb_{ira}}$$

Where *PD*_*ira*_ is percentage difference in the mortality rate, *MRb*_*ira*_ = *MRb (L*_*i*_ *E*_*r*_ *Aa)* is the rates of cervical cancer death, among Blacks and Whites for geographic area *i,* race *r* and age group *a* respectively; and $P20_{ira}^{l}=P20(L_{i}E_{r}A_{a})$; for *l =0, 1 is* the Healthy People 2020 US baseline and target mortality rate respectively. The indices *i = 1, 2 is Alabama and the US, r = 1, 2 is* Blacks and Whites*, a = 1, 2* is age group 20–64 years and 65 years & older respectively.

## 3. Results

### 3.1 Disparity in Cervical Cancer Screening Percentages by Race and Age

Between 2000 and 2010, in comparison and controlling for age, in Alabama cervical cancer screening percentages of Blacks were on average 87.65% always higher than those of Whites, and slightly below the national average of 87.85% which was also higher in Blacks. Controlling for race, cervical cancer screening percentages among Alabamian women,18 years and older and 65 years and older was on average 84.87% and 71.6% respectively, higher than their women counterparts at the national levels with the average of 84.3 % and 69.92% respectively (Table 4 in Appendix).

**Figure 1.**
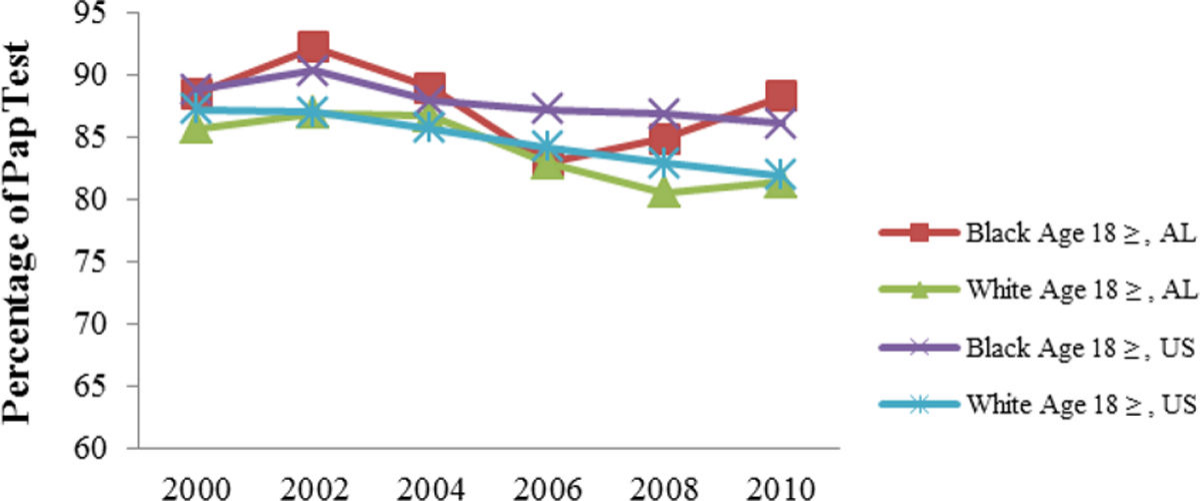
Cervical cancer screening percentages of Blacks and Whites aged 18 years and older in Alabama and the US

Figure 1 shows utilization of cervical cancer screening among Black and White Alabamians from 2000 to 2010 in comparison to the national data. Black Alabamians, 18 years and older had been utilizing cervical cancer screening at a slightly higher rate than their White counterparts at the State and national levels. However, the rate has been fluctuating from 83.0% in 2006 to a peak of 88.3% in 2010. Utilization of cervical cancer screening by Black women at the national level followed similar trend

### 3.2 How Far Away in Percentage From the Healthy People 2020 US Baseline and Target Is Cervical Cancer Screening in Blacks and Whites

**Figures 2 A & B.**
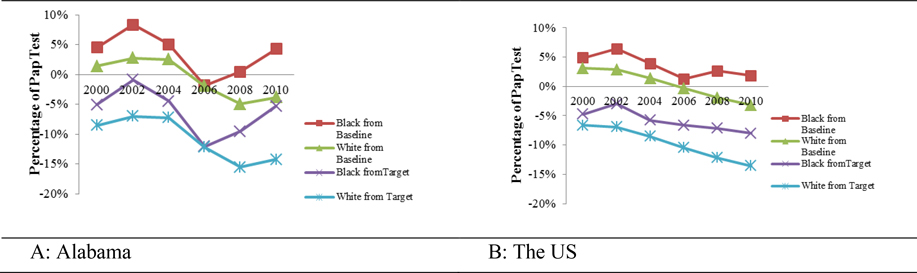
How far away in percentage from the Healthy People 2020 US baseline and target is cervical cancer screening in Blacks and Whites aged 18 years and older in Alabama and the US

Figures 2 A and B illustrate the utilization of cervical cancer screening by Blacks and Whites, 18 years and older, in Alabama and the US in comparison to the US baseline (84.5%) and the target (93.3%) set by Healthy People 2020 (Table 1). The data show that Blacks utilize cervical cancer screening better than their White counterparts at both the State and national levels. Compared to the US baseline, utilization of cervical cancer screening by Blacks in Alabama fluctuated from 2000 to 2010, reaching the peak of 8% above the US baseline in 2002 and dropping to 2% below the US baseline in 2006. Compared to the US target, cervical cancer screening by Blacks in Alabama similarly fluctuated from 2000 to 2010, reaching the peak of 1% below the US target in 2002 and dropping to 12% below the US target in 2006. The trends for White Alabamians followed the same pattern: reached a peak of 3% above the US baseline in 2002 and 2004 and dropped below it by 5% in 2008. Similarly, the US target for the utilization of cervical cancer screening by White Alabamian also fluctuated from 2000 to 2010, reaching the peak of 7% below the US target in both 2002 and 2004 and dropping further to 16% below the US target in 2008. At the national level, utilization of cervical cancer screening by Blacks fluctuated between 2000 and 2010. Compared to the US baseline, screening percentages reached the peak of 6% in 2002 and dropped to 1% above the US baseline in 2006. In contrast, the trends for Whites declined from a peak of 3% above the US baseline in 2000 and 2002 to 3% below the US baseline in 2010. Compared to the US target, utilization of cervical cancer screening by Blacks nationally peaked at 3% below the US target in 2002 and dropped further to 8% below the US target in 2010. On the other hand, cervical cancer screening by Whites nationally dropped continuously from 7% below the US target in 2000 and 2002 to 14% in 2010.

### 3.3. Cervical Cancer Mortality Rates by Race and Age

From 2002 to 2012, in comparison, in Alabama cervical cancer mortality rates of Blacks 65 years and older was on average 22.3% which was significantly higher, more than tripling that of Blacks, 20 – 64 and Whites, 20 – 64 and 65 years and older who on average had 4.03%, 3.07% and 5.12% respectively. At the national levels, the average mortality rates due to cervical cancer were slightly below the State level. Blacks, 65 years and older had a higher rate on average 13.9%, compared to Blacks, 20 – 64 years old and Whites, 20 – 64 and 65 years and older who on average had 4.23%, 2.57%, and 5.28% respectively (Table 5 in Appendix).

**Figures 3 A & B.**
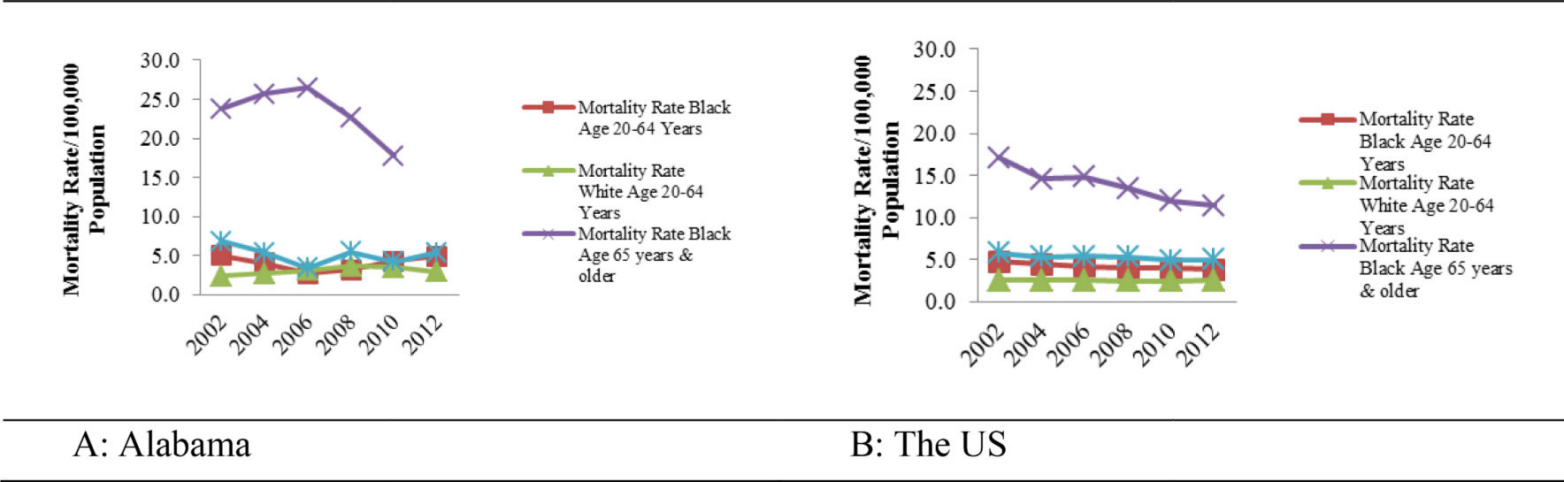
Cervical cancer mortality rates per 100,000 populations of women aged 20 – 64 and 65 years and older in Alabama and the US

Table 5 (in Appendix) and Figure 3 – A show that that during 2002, 2004, 2010 and 2012, the rate of cervical cancer mortality for Black Alabamians, 20 – 64 years old, and was almost double of that of their White counterparts. In this group mortality ranged from 4.1 in 2004 to 5.0 in 2002 and from 4.3 to 4.9 per 100,000 population. Black Alabamians, 65 years and older however, die due to cervical cancer at consistently higher rates (three times higher than their White counterparts). In this group mortality ranged from 2.7 in 2006 to 5.0 in 2002 per 100,000 population. Black Alabamians, aged 65 years and older however, die due to cervical cancer at consistently higher rates (three times higher than their White counterparts). From 2002 to 2012, Blacks, 20 – 64 years old across the US have almost double cervical cancer mortality rate compared to their White counterparts [(Table 5 (in Appendix) and Figure 3 – B)] although mortality declined between 2002 and 2012 (from 4.8 to 3.8 per 100,000 population). Consistent to the State level data, nationwide cervical cancer mortality rate in Blacks, 65 years and older was three times higher than that of their White counterparts.

### 3.4 Trends in Cervical Cancer Mortality Rates

**Figures 4 A & B.**
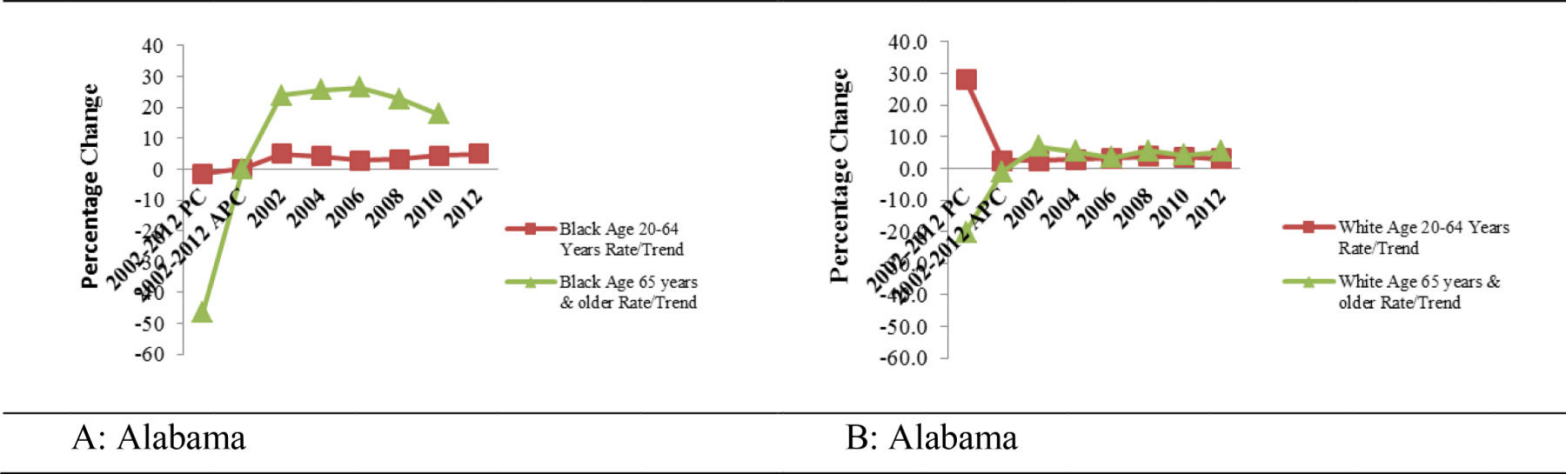
Trends in age-adjusted cervical cancer mortality rates of Blacks and Whites in Alabama. Specifically, the calculated Percentage Changes (PC) and Annual Percentage Changes (APC) for the entire time 2002 – 2012

Table 3 and Figures 4 – A and B depict the trends in cervical cancer mortality in Alabama from 2002 and 2012. Overall, cervical cancer mortality among Blacks aged 20 – 64 years old decreased by 1.5% although a 46.4% reduction in mortality was recorded for Blacks aged 65 years and older, between 2002 and 2012. In contrast, cervical cancer mortality in Whites aged 20 – 64 years old increased by 28% (APC = 2.5%) from 2002 through 2012. However, during the same period, the mortality declined by 20.3% (APC = −1.2 %) in Whites aged 65 years and older.

**Figures 5 A & B.**
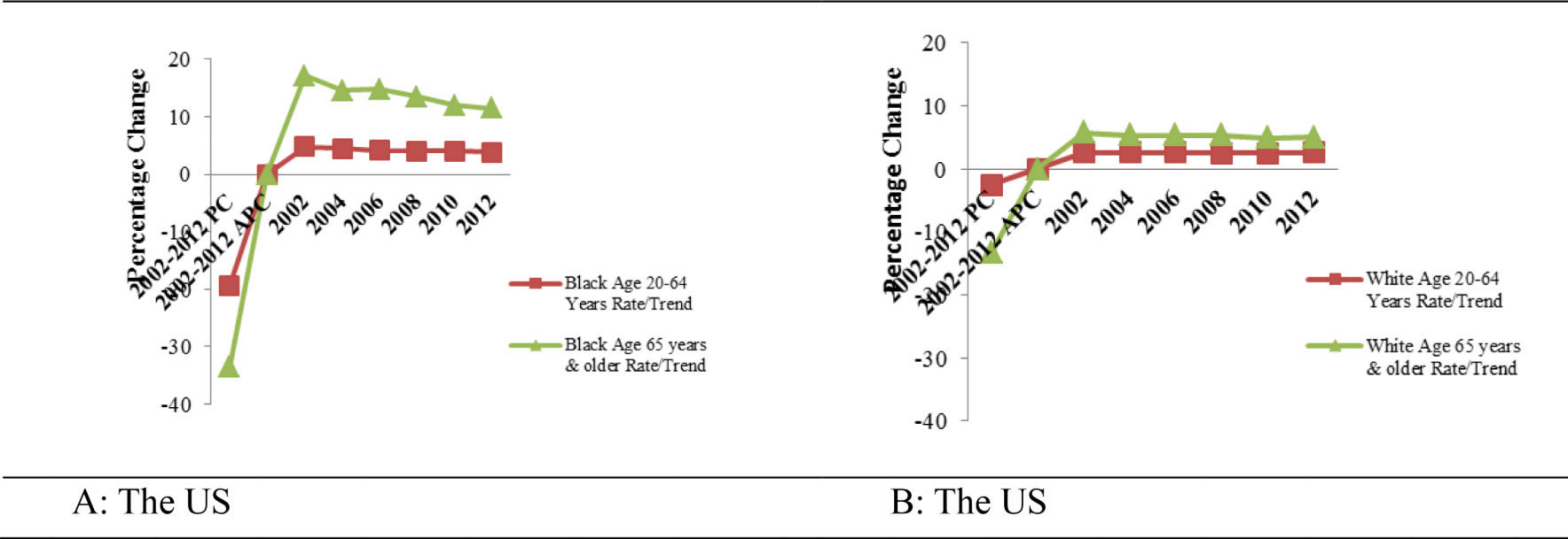
Trends in age-adjusted cervical cancer mortality rates of Blacks and Whites in the US. Specifically, the calculated Percentage Changes (PC) and Annual Percentage Changes (APC) for the entire time 2002 – 2012

Trends in the national cervical cancer mortality rates from 2002 to 2012 are presented in Table 3 and Figures 5 – A and B. Overall, cervical cancer mortality among Blacks aged 20 – 64 years old decreased by 19.3%, with significant APC (−1.8%; *p < 0.05*). For Black women aged 65 years and older, cervical cancer mortality decreased by 33.5%, with significant APC (−3.4%; p < 0.05). Similarly, cervical cancer mortality among Whites, aged 20 – 64 years old decreased by 2.5%. The APC was significant (−0.5%; *p < 0.05*). On the other hand, Whites aged 65 years and older, had an overall decrease of 13.3% (APC = −1.2%; *p < 0.05*).

### 3.5 Percentage Difference in Cervical Cancer Mortality From the US Baseline and Target Rates

**Figures 6 – A & B.**
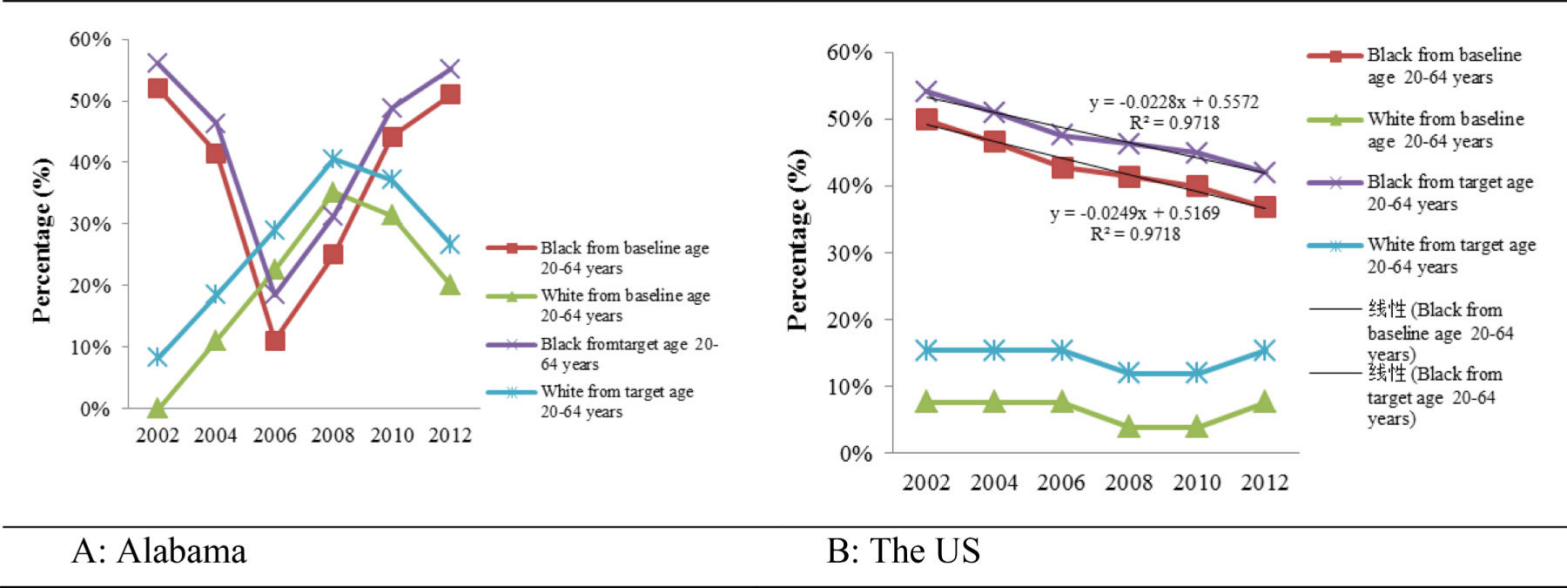
The percentage differences in cervical cancer mortality rates of Blacks and Whites aged 20 – 64 years in Alabama and the US compared to the Healthy People 2020 US baseline and target rates

Figures 6 – A and B show the PD in cervical cancer mortality rate of Blacks and Whites in Alabama and the US compared to the US baseline (2.4) and target (2.2) mortality rates set by Healthy People 2020 (Table 3). For Black Alabamians aged 20 – 64 years old, cervical cancer mortality rate exceeded the US baseline and target mortality rates respectively by an average of 37% (11% - 52%) and 43% (19% - 56%), from 2002 to 2012. In comparison, cervical cancer mortality rate of White Alabamians was on average 20% (0 – 35%) below the US baseline or exceeded target mortality rate by an average of 27% (8% - 41%) from 2002 to 2012. At the national level, cervical cancer mortality rates in Blacks of the same age group exceeded the US baseline and target by more than 30%, while Whites only exceeded the US baseline by less than 10% and target by less than 20%.

**Figures 7 – A & B.**
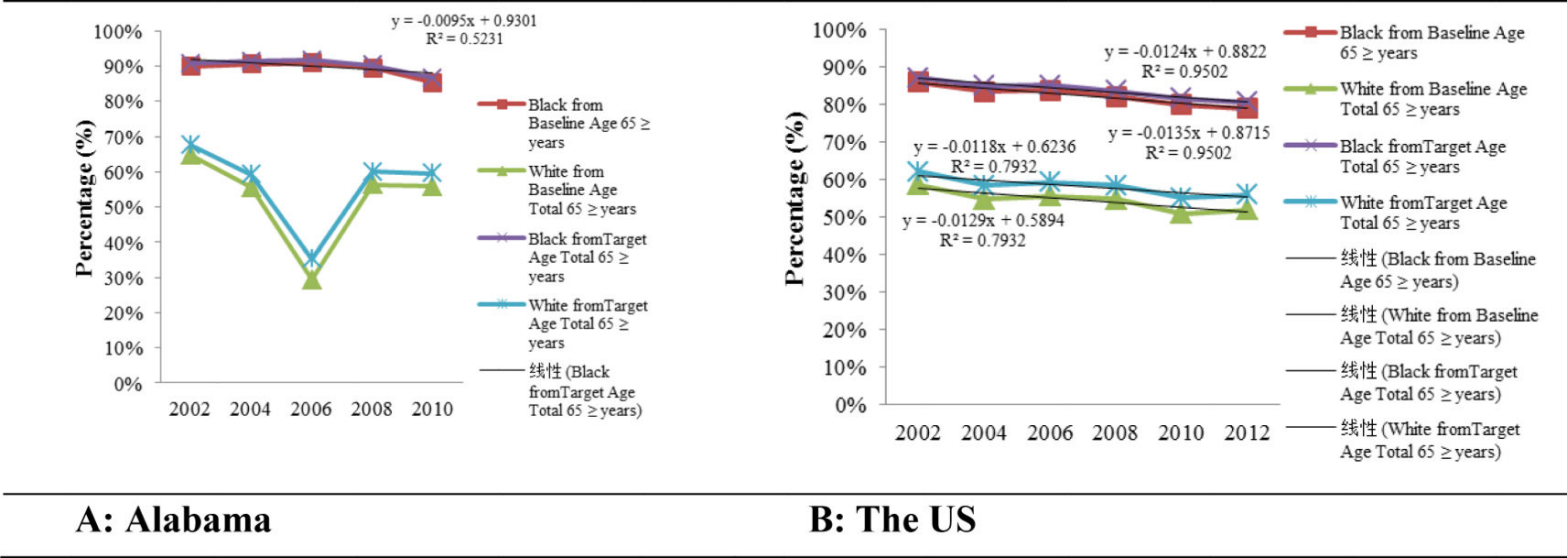
The percentage differences in cervical cancer mortality rates of Blacks and Whites aged 65 years and older in Alabama and the US compared to the Healthy People 2020 US baseline and target rates

Likewise, Figures 7 – A and B illustrate the percentage difference in cervical cancer mortality rates of Blacks and Whites, aged 65 years and older, in Alabama and the US compared to the US baseline (2.4 per 100,000 population) and target (2.2 per 100,000 population) mortality rates set by Healthy People 2020 (Table 2). At the State and national levels, cervical cancer mortality rates in Blacks exceeded the US baseline and target rates by more than 80%; while, in Whites it was always below 70%.

## 4. Discussion

Using the 1998 US rate of 3.0 deaths per 100,000 population as the US baseline, Healthy People 2010 called for a reduction in cervical cancer mortality to 2 deaths per 100,000 populations by 2010. In 2005, women across the US made encouraging progress towards this goal by achieving a rate of 2.5 per 100,000 population (3). The rate further declined to 2.4 per 100,000 population between 2006 and 2010. In contrast, Alabama has not seen a significant decline in cervical cancer mortality rate (3.0 per 100,000 population) in recent years. Furthermore, Blacks in Alabama have a higher cervical cancer mortality rate (5.3 per 100,000 population) than Whites (2.4 per 100,000 population) (Alabama Statewide Cancer Registry (ASCR) & Alabama Department of Public Health, 2012). As a result, the initiatively established goal to reduce the number of new cases, including illness, disability, and death caused by cancer, through an increased use of preventive measures, had not been met by the year 2010. Using the 2007 US rate of 2.4 deaths per 100,000 population as the US baseline, Healthy People 2020 calls for a reduction in cervical cancer mortality to 2.2 deaths per 100,000 population by 2020 (C. f. D. C. a. P. (CDC), 2014). In the process, Healthy People 2020 kept the same goal of Healthy People 2010 for all cancers. However, all the cervical cancer objectives have been expanded, modified and/or archived to include a broader range of measures reflecting the latest trends in cancer prevention and diagnosis (Tables 1 and 3).

The University of Alabama at Birmingham (UAB) Comprehensive Cancer Center’s Deep South Network (DSN) for Cancer Control now includes research among its core outreach and awareness activities to reduce cancer disparities in the South’s poorest communities. Cancer rates in minority and under-served populations in the South are among the nation’s highest. The DSN sponsored by National Cancer Institute, for more than 10 years, the DSN has targeted two poor, rural regions Alabama’s Black Belt and the Mississippi Delta and the urban areas in Jefferson County, Alabama, and the Hattiesburg/Laurel, Mississippi, metropolitan area. The DSN has been effective in raising cancer awareness, improving both education and outreach to its target populations, and increasing the use of cancer screening services. The goal of eliminating cancer health disparities will be pursued in the targeted rural and urban counties in Mississippi and Alabama using Community-Based Participatory Research (Lisovicz N1 et al., 2006). A study conducted in Mississippi, showed that after screening for cervical cancer, the cervical cancer mortality rates for both races had decreased slopes during 1975 to 2010. Comparing cervical cancer mortality rates at the national level, Mississippi had the fifth highest rate for Blacks and the eleventh highest for Whites. Also for the declining cervical cancer mortality rate slopes at the national level, Mississippi had the sixth highest rate for both Blacks and Whites (Tabatabai MA et al., 2014). For both decreases in cervical cancer mortality rates because of screening, this is in line with the “Healthy People 2010 and 2020 goal”, those of targeting reduction in cervical cancer mortality rates from the US baseline. In our study, we found the contrary in that instead of expecting a decrease a decrease in cervical cancer mortality rate after screening, there was instead an increase in cervical cancer mortality rate in fact, more than tripling in Blacks than their Whites counterparts in Alabama. Interestingly, the relationship between Black and White Mississippians is reversed when considering recent Pap testing. The disparities in cervical cancer mortality rate at the national level in Whites compared to Blacks in both races are much larger in magnitude. Disparities between Mississippian Whites, and Blacks the magnitude is even much larger (National Cancer Institute (NIH), 2015). In contrast, in this study our results showed high rates of cervical cancer mortality among Black Alabamians, despite high screening percentages compared to White Alabamians. Furthermore, the disparities between Alabamian Blacks, US Blacks, Alabamian Whites, and the US Whites are much larger in magnitude.

Complex and interrelated factors contribute to the observed significantly high cervical cancer mortality rates in Blacks and underserved Whites. The most obvious factors are associated with a lack of health care coverage and low socioeconomic status (SES). Furthermore, adherence to follow-up after an abnormal Pap test is also low among minority groups (Engelstad et al., 2001), (Cardin et al., 2001). In line with this, the NBCCEDP, followed over 10,000 participants with two Pap tests of abnormal squamous cells of undetermined significance or low-grade intraepithelial lesions. Only 44% were followed-up according to established guidelines. According to the report, Blacks had a higher percentage of no follow-up compared with other racial or ethnic groups (Benard, Lawson, Eheman, Anderson, & Helsel, 2005). In addition to a low rate of follow-up, a marked difference in treatment is another potential explanation as Blacks are less frequently treated for cervical cancer or are treated inappropriately. The study indicated that, among patients who have cervical cancer-directed surgery as part of their primary treatment, significantly more Blacks have local, non-radical surgery compared with Whites, despite the fact that Blacks have the greatest proportion of later stages (Stage 2, 3, and 4) (43%) tumors than Whites (34%) (Patel et al., 2005). However, reviewed data in another previous study (Del Carmen, 1999) of over 2300 women, indicated that Blacks over 35 years of age diagnosed with stage 1A (2) cervical cancer were less likely to be treated with a hysterectomy than their White counterparts. Another disparity of cervical cancer high mortality in Blacks is that comparatively to the Whites, a greater percentage of Blacks have no surgery, have radiation therapy only, or have no therapy as their first treatment after a cervical cancer diagnosis. The disparities in survival may be partially attributed to racial/ethnic variations in prognostic factors for cervical cancer, such as co-morbid conditions (Del Carmen MG et al., 1999). Other factors that may contribute to disparities in treatment for Blacks include poorer health, patient’s refusal of treatment, and lack of physician recommendation for treatment (Shavers & Brown, 2002). Summing up, after adjusting for age at diagnosis, histology, stage, and first course of treatment, Hispanic patients had a 26% decreased risk of death from invasive cervical cancer compared with non-Hispanic patients, while Black patients had a 19% increased risk of death (Downs LS, Smith J S, Scarinci I, Flowers L, & Parham G, 2008).

Our recent study shows that in urban, rural Black Belt (BB) and other rural counties of Alabama, between 2004 and 2013, racial variations in treatment were found between Black and White patients. A disproportionate number of cervical cancer deaths occur among racial/ethnic minorities, particularly Blacks than Whites. Although differences in prevalence rate and stage of disease at diagnosis may contribute to racial disparities in mortality, evidence of racial disparities in the receipt of treatment of other chronic diseases raises questions about the possible role of inequities in the receipt of cancer treatment (Abdalla E, 2017). Our study indicated that surgical treatment for Black patients who were diagnosed with cervical cancer in rural BBC was significantly less common amongst Black than White patients. Differences in care may contribute to racial disparities in outcomes for women with cervical cancer. Monte Carlo simulations for generating probability distributions for the likelihood analysis was done for the assessment of cervical cancer prevalence rate in urban, rural BB and other rural counties of Alabama. This method allows for the use of data coming from ADPH, Cancer Registry and the enhanced surveillance aimed at treating Black patients living in these counties. The procedure provides the necessary input to the large-scale computational model for the analysis of different treatments as mitigations in the future (Abdalla E, 2017).

A previous study examined associations of cervical cancer with age and other covariates, stratified by insurance type. Although privately insured patients had lower percentages of advanced-stage disease across all age groups, they also had the steepest gradient of increasing relative risk by age of all insurance groups. Among privately insured women, an approximately tripled relative risk for women aged 55 years and older was found; among women in other insurance categories, maximum relative risks were around two. Among uninsured, Medicaid-insured, and Medicare-insured patients, Hispanics and women of other races were less likely to be diagnosed with late-stage disease than were Whites. Among privately insured and older Medicare-insured women, Blacks were more likely than Whites to be diagnosed with late-stage disease. Patients with adenocarcinomas were less likely than women with squamous cell carcinoma (SCC) to be diagnosed with late-stage disease, regardless of insurance type (Fedewa et al., 2012).

Increasing the proportion of uninsured and Medicaid-insured cervical cancer patients diagnosed at an earlier stage through improved screening and follow-up after an abnormal result is not only important to lower morbidity and mortality, but may also offer cost savings, because advanced-stage cervical cancer is more expensive to be treated than early-stage cancer (Subramanian et al., 2010). Although education by zip code area was adjusted, individual SES, such as lack of transportation and logistical challenges (time off work and child care), may vary by age and negatively influence screening among women with access to care (Daley et al., 2011). Other studies have reported older age as the most prominent predictor of failure to screen among women within comprehensive health insurance plans (Leyden et al., 2005), highlighting the need for increased Pap testing adherence even among middle-aged women with access to care for whom it is recommended (Saslow et al., 2002). A previous study identified that ideal distance for cancer patients to the hospital should be less than 12.4 miles away to avoid pain and discomfort during the travel (Malaysian Oncology Society, 2012). Our recent study shows that out of seven sexually transmitted diseases (STD) clinics, nine health care centers and six hospitals are in Macon County, and nearby Alabama counties. There is only one health care center, namely Macon County Health Department, which has an ideal location (around 1.9 to 2.5 miles from Macon County). However, it provides only Pap smear but no treatment (Abdalla E, 2017).

In the US between 2008 and 2011, our recent study shows that there is a correlation between age, race, income and comorbid illnesses (e.g. obesity, diabetes, hypertension and chronic pulmonary disease) and mortality rates of cervical cancer in Black women. Significant associations were observed between the cervical cancer among Blacks and Whites, who were obese and not obese by the annual income only in 2008. In 2009 and 2011, a significant association was observed between the different types of insurance among Blacks and Whites, who were obese and not obese and were diagnosed with cervical cancer (Abdalla E, 2017). We observed that Blacks, who were obese and not obese, were more likely to have Medicaid. Previous studies reported that SES (such as income), race had been identified as some of factors related to increase the likelihood of cervical cancer patients. In the large, all - payer, nationwide database of hospitalized patients, we compared the level of income among Black obese and not obese patients, who were diagnosed with cervical cancer. We observed that Black obese and not obese patients, with low household income were more likely to be diagnosed with cervical cancer. The presence of comorbid illness may be independent and additive in the determination of cervical cancer prognosis. Such comorbidity may be particularly significant for obese Black women (Abdalla E, 2017).

To evaluate the trends during 2002 – 2012, we assessed cervical cancer mortality rates in Blacks and Whites in Alabama and the US. An overall decrease in Cervical cancer mortality (PC= −1.5) was recorded for Blacks compared to Whites, with significant APC at the national level although there was no any change in the APC during that time at the State level. The differences detected in this sample are likely to be magnified in rural and other medically underserved and poor populations. The American Hospital Association (AHA) data include non-ACoS hospitals; these hospitals are more likely to be smaller and have fewer fulltime medical personnel. They more often are in rural counties with low income and educational attainment, and in counties with higher cervical cancer mortality. They are also less likely to have oncology and radiation services. Eighteen percent of all US counties have no hospital, and Cervical cancer mortality is markedly higher in these counties compared to those with hospitals (H. P. a. W. Freeman, B.K., 2005).

To assess the relationship between a high cervical cancer mortality rate and decreasing trends in Blacks we analyzed the PD in cervical cancer mortality rate of Blacks and Whites in Alabama and the US in comparison to the US baseline (2.4 deaths per 100,000 population) and target (2.2 deaths per 100,000 population) mortality rates set by Healthy People 2020. For Blacks, cervical cancer mortality rate exceeded both the US baseline and target rates between 2002 and 2012, whereas, for Whites the rates were below the US baseline although they exceeded the target rates. Previous studies stated that screening rates were higher among younger women compared with older women (H. P. a. W. Freeman, B.K., 2005). Early detection through screening is a factor in increasing the pace of decline in cervical cancer mortality; direct consequences are the reductions in mortality rates for both races and a reduction of racial disparities (Tabatabai MA et al., 2014). Contrary, our results illustrate that the disparity of cervical cancer mortality rates between Blacks and Whites at the State and national levels exists. Between 2002 and 2012 and despite rigorous screening, the mortality rates of cervical cancer in Alabama doubled and tripled among Black women ages 20 – 64 and 65 years and older respectively compared to White women.

Since disparities in cervical cancer treatment practices and follow-up rates among minority populations may lead to a higher mortality, many projects have been launched at the Federal, State, and local levels to determine effective methods of reducing these disparities (H. P. a. W. Freeman, B.K., 2005). Federally funded initiatives, such as the NBCCEDP as mentioned previously, provide funding for screening and programs that increase awareness in target communities (T. C. f. D. C. a. P. (CDC), 2015), (Ryerson, 1991–2002). For cancer patients to receive timely diagnosis and treatment, the Patient Navigator Academy, a program from the National Cancer Institute (NCI), uses patient navigators to overcome barriers to care and helps patients “navigate” the healthcare system. Navigators represent a wide range of racial and ethnic populations from different geographical locations. Patient navigators are involved in scheduling appointments, coordinating insurance, community outreach, forming partnerships in the community, and encouraging clinical trial participation (Fowler, Steakley, Garcia, Kwok, & Bennett, 2006).

Community partnerships have been established across the country. The Special Populations Networks (SPNs) initiative, established by the NCI in 2000, aims to reduce cancer disparities by educating healthcare providers and community leaders and providing community outreach programs (H. P. Freeman & Vydelingum, 2006). SPNs include five national, two regional, and 11 local programs the major goal was to promote cancer awareness research in the minority and underserved communities, targeting most minority populations. The utilization of lay healthcare workers in community outreach programs and telephone counseling, and the availability of culturally-appropriate educational materials have all been shown to improve screening and follow-up rates among minority women (Wasserman, Bender, & Lee, 2007). Newly developed prophylactic vaccines, which target the most prevalent oncogenic HPV types responsible for approximately 77% of all cervical cancer and 54% of high-grade precancerous lesions in the US, are well-tolerated and effective (Smith et al., 2007). Prophylactic vaccines can provide primary prevention against cervical cancer and strategies employed to enhance screening and follow-up rates can be extended to promote vaccination (Leyden WA, Manos MM, & Geiger AM. et al, 2005).

## 5. Conclusions

In conclusion, previous studies have revealed that there is a relationship in cervical cancer screening and mortality rates between Blacks and Whites, the relationship being that the more you screen for cervical cancer the higher we expect a decrease in cervical cancer mortality rate. Therefore, the results of the present study show it conclusively that in Alabama a disparity still exists for the high cervical cancer mortality in Blacks despite the higher percentages of screening for cervical cancer as would otherwise be expected. Additionally, the state has not yet achieved the Healthy People 2020 goal. Thus, although the gap in mortality rates in Alabama has narrowed, however the disparity remains, but if the dismal narrowing is sustained, it will reduce the racial disparities in cervical cancer mortality though at a very slow rate. Therefore, Public health officials should monitor progress toward reduction and/or elimination of these disparities.

## Figures and Tables

**Table 1.** Comparison between healthy people 2010 and healthy people 2020 cervical cancer screening’s goal, objective, the US baseline and target percentages

	Healthy People 2010	Healthy People 2020
Goal	Reduce the number of new cancer cases as well as the illness, disability, and death caused by cancer	Reduce the number of new cancer cases, as well as the illness, disability, and death caused by cancer
Objective	3–11 Increase the proportion of women who receive a Pap test: 3–11a: Women aged 18 years and older who have ever received a Pap test. 3–11b: Women aged 18 years and older who received a Pap test within the preceding 3 years.	C-15 Increase the proportion of women who receive a cervical cancer screening based on the most recent guidelines
Baseline	For objective 3–11a: 92.0 percent For objective 3–11b: 79.0 percent	84.5 percent of females aged 21 to 65 years received a cervical cancer screening based on the most recent guidelines in 2008 (age adjusted to the year 2000 standard population)
Target	For objective 3–11a: 97.0 percent For objective 3–11b: 90.0 percent	93.0 percent
Target-Setting Method	Better than the best	10 percent improvement
Data source	National Health Interview Survey (NHIS), CDC, NCHS	National Health Interview Survey (NHIS), CDC/NCHS
Data	**Healthy People 2010:** The HP2010 objective with the same definition was 03–11b. http://wonder.cdc.gov/data2010/	**Data 2020:** http://www.healthypeople.gov/2020/data-search/Search-the-Data?nid=4053 **Spotlight on Disparities:** • Race/Ethnicity • Educational Attainment **Data Details:** http://www.healthypeople.gov/node/4053/data_details

**Table 3.** Trends in age-adjusted cervical cancer mortality rates of Blacks and Whites in the US. Specifically, the calculated Percentage Changes (PC) and Annual Percentage Changes (APC) for the entire time 2002 – 2012

	20–64 years old								65 years and older							
	
Years of Death	Black				White				Black				White			
	
Alabama	Rates/Trends	SE	Lower CI	Upper CI	Rate/Trends	SE	Lower CI	Upper CI	Rate/Trends	SE	Lower CI	Upper CI	Rate/Trends	SE	Lower CI	Upper CI

2002–2012 PC	−1.5				28.0				−46.4				−20.3			

2002–2012 APC	0.0		−4.8	5.0	2.5		−0.6	5.7	∼		∼	∼	−1.2		−7.0	4.9

2002	5.0	1.2	2.9	8.0	2.4	28.0	1.5	3.6	23.8	5.8	13.9	38.3	6.8	1.6	4.1	10.6

2004	4.1	1.1	2.3	6.8	2.7	2.5	1.8	4.0	25.7	6.1	15.2	40.6	5.4	1.4	3.0	8.8

2006	2.7	0.9	1.3	5.0	3.1	2.4	2.1	4.4	26.5	6.1	15.9	41.5	3.4	1.1	1.6	6.3

2008	3.2	0.9	1.6	5.5	3.7	2.7	2.6	5.1	22.7	5.7	12.9	36.8	5.5	1.4	3.1	8.9

2010	4.3	1.0	2.5	6.8	3.5	3.1	2.4	4.8	17.75	^	^	^	4.2	1.2	2.2	7.4

2012	4.9	1.2	2.9	7.7	3.0	3.7	2.1	4.3	12.8	4.1	6.1	23.7	5.4	1.3	3.1	8.6

**US**																

2002–2012 PC	−19.3				−2.5				−33.5				−13.3			

2002–2012 APC	−1.8*		−2.3	−1.2	−0.5*		−0.8	−0.1	−3.4*		−4.2	−2.6	−12*		−1.9	−0.4

2002	4.8	0.2	4.4	5.2	2.6	0.1	2.5	2.8	17.1	1.0	15.3	19.1	5.8	0.2	5.5	6.2

2004	4.5	0.2	4.1	4.9	2.6	0.1	2.5	2.7	14.6	0.9	12.9	16.4	5.3	0.2	5.0	5.7

2006	4.2	0.2	3.8	4.5	2.6	0.1	2.5	2.7	14.8	0.9	13.1	16.6	5.4	0.2	5.1	5.8

2008	4.1	0.2	3.8	4.5	2.5	0.1	2.4	2.7	13.5	0.8	12.0	15.2	5.3	0.2	5.0	5.7

2010	4.0	0.2	3.7	4.4	2.5	0.1	2.4	2.6	12.0	0.8	10.6	13.5	4.9	0.2	4.6	5.3

2012	3.8	0.2	3.5	4.2	2.6	0.1	2.5	2.7	11.4	0.7	10.0	12.9	5.0	0.2	4.7	5.3

Underlying mortality data provided by NCHS (www.cdc.gov/nchs).

Rates are per 100,000 and age-adjusted to the 2000 US Std Population (19 age groups - Census P25-1130) standard; Confidence intervals (CI) are 95% for rates (Tiwari mod) and trends.

Percent changes were calculated using 1 year for each end point; APCs were calculated using weighted least squares method. Statistic could not be calculated. Statistic not displayed due to fewer than 10 cases.

The APC is significantly different from zero (p<0.05).

The red bolded Rate/Trend is the average of Rates/Trends of 2008 and 2012 because in 2010 the Statistic not displayed due to fewer than 10 cases.

## Appendix

**Table 4.** Cervical cancer screening percentages by Blacks and Whites aged 18 years and older, in Alabama and the US, 2000 – 2010

Location	Year	Pap Test in the Past 3 Years (%)			
		Blacks	Whites	Total, Women 18 Years and Older	Total, Women 65 Years and Older
**Alabama**	2000	88.5	85.7	86.6	72.6
	2002	92.2	86.9	88.2	77.1
	2004	89.0	86.7	87.2	72.7
	2006	83.0	82.9	82.7	74.8
	2008	84.9	80.5	81.3	63.8
	2010	88.3	81.4	83.2	68.6
	**Average**	87.65	84.02	84.87	71.60

**US**	2000	88.8	87.2	86.8	74.4
	2002	90.3	87	86.2	74.4
	2004	87.9	85.7	85.2	71.2
	2006	87.2	84.2	83.7	70.8
	2008	86.8	82.9	82.8	65.8
	2010	86.1	81.9	81.1	62.9
	**Average**	87.85	84.82	84.30	69.92

Data Source: Behavioral Risk Factor Surveillance System, Centers for Disease Control and Prevention.

**Table 5.** Alabama and the US cervical cancer mortality rates, by Race, and age group, 2002–2012

Location	Year	Mortality Rates			
		Black Age 20–64 years	White Age 20–64 years	Black Age 65 ≥ years	White Age 65 ≥ years
**Alabama**	2002	5.0	2.4	23.8	6.8
	2004	4.1	2.7	25.7	5.4
	2006	2.7	3.1	26.5	3.4
	2008	3.2	3.7	22.7	5.5
	2010	4.3	3.5	^	4.2
	2012	4.9	3.0	12.8	5.4
	**Average**	4.03	3.07	22.30	5.12

**US**	2002	4.8	2.6	17.1	5.8
	2004	4.5	2.6	14.6	5.3
	2006	4.2	2.6	14.8	5.4
	2008	4.1	2.5	13.5	5.3
	2010	4	2.5	12	4.9
	2012	3.8	2.6	11.4	5
	**Average**	4.23	2.57	13.90	5.28

Rates are per 100,000 and age-adjusted to the 2000 US (19 age groups) standard.

Data Source: The Surveillance, Epidemiology, and End Results (SEER).

## Abbreviations

MR: Mortality Rates
PD: Percentage Difference
PC: Percentage Change
APC: Annual Percentage Change
CDC: Centers for Disease Control and Prevention
BRFSS: Behavioral Risk Factor Surveillance System
SEER: Surveillance, Epidemiology, and End Results
HPV: Human Papillomavirus
Pap test: Papanicolaou Test
NBCCEDP: National Breast and Cervical Cancer Early Detection Program
USPSTF: United States Preventive Services Task Force
SCC: squamous cell carcinoma
NHIS: National Health Interview Survey
NCHS: National Center for Health Statistics
SES: Socioeconomic Status
ACoS: American College of Surgeons
NCI: National Cancer Institute
SPNs: Special Populations Networks
NCDB: National Cancer Data Base
AHA: American Hospital Association
